# Neoadjuvante Chemotherapie beim operablen Kolonkarzinom – ein potenzieller neuer Standard?

**DOI:** 10.1007/s00066-023-02161-y

**Published:** 2023-10-10

**Authors:** Georg W. Wurschi

**Affiliations:** 1 Universitätsklinikum Jena, Klinik für Strahlentherapie und Radioonkologie, Friedrich-Schiller-Universität Jena, 07747 Jena, Deutschland; 2 Universitätsklinikum Jena, Interdisziplinäres Zentrum für Klinische Forschung (IZKF), Clinician Scientist-Programm (CSP), Friedrich-Schiller-Universität Jena, 07747 Jena, Deutschland

## Hintergrund

Die neoadjuvante Therapie ist bei verschiedenen gastrointestinalen Tumoren eine effektive Möglichkeit zur Steigerung der Tumorkontrolle und des Downstagings. Beim Kolonkarzinom ist sie jedoch bisher kein Standard.

## Patienten und Methodik

In der randomisierten FOxTROT-Studie wurden 1053 Patienten aus 85 Zentren im 2:1-Design zwischen neoadjuvanter Chemotherapie (NAC+OP, Gruppe A) und primärer Op. mit adjuvanter Chemotherapie (AC, Gruppe B) aufgeteilt. Eingeschlossen wurden Patienten mit T3/T4-Tumoren (davon 75 % mit N +) und extramuraler Gefäßinvasion (EMVI+), die als fit für Chemotherapie und Op. galten. In beiden Gruppen kam FOLFOX oder alternativ CapOx zur Anwendung; Gruppe A erhielt 3 Zyklen NAC sowie 9 (nach späterem Amendment auch nur noch 3) Zyklen AC; in Gruppe B waren 12 (später 6) Zyklen AC vorgesehen.

Dabei erhielten Patienten mit RAS-wild-type-Tumoren 1:1-randomisiert Panitumumab zusätzlich zur Chemotherapie oder nicht. Dies wurde in einem späteren Amendment auf die NAC-Gruppe A beschränkt. Die Indikation zur AC wurde mit Randomisierung unabhängig vom histologischen Ergebnis bei Resektion gestellt. Der primäre Endpunkt war die Rezidivrate (lokal/„distant“) nach 2 Jahren, die gesamte Beobachtungszeit betrug 5 Jahre.

## Ergebnisse

Patienten in der mit NAC behandelten Gruppe A hatten signifikant weniger Rezidive oder residuale Krankheitsaktivität nach 2 Jahren (16,9 %) als in der Kontrollgruppe B (21,5 %, HR 0,72, *p* = 0,037). Die krankheitsspezifische Mortalität und Gesamtmortalität wurden ebenfalls mit vergleichbarer Hazard Ratio gesenkt (HR 0,74 bzw. 0,76; der absolute Unterschied betrug nach 5 Jahren 6 bzw. 4 Prozentpunkte); diese Werte erreichten aber keine Signifikanz. Die Todesrate war in der Kontrollgruppe nach 5 Jahren leicht erhöht, wenn auch nicht signifikant. In der NAC-Gruppe A kam es erwartungsgemäß zu einem signifikanten Downstaging, aber auch zu einer signifikant größeren Rate an R0-Resektionen (94 % vs. 89 %). Es zeigten sich weniger perioperative Komplikationen in der NAC-Gruppe A als in der AC-Gruppe B. Die Hinzunahme von Panitumumab verbesserte die Ergebnisse in der NAC-Gruppe nicht. Interessanterweise wurde bei Patienten mit Mikrosatelliten-instabilen Tumoren (MSI) in dieser Studie eine geringere Remissionsrate nach NAC beobachtet als bei Mikrosatelliten-stabilen (MSS) Tumoren (7 % vs. 23 %, *p* < 0,01); das 2‑Jahres-Rezidivrisiko wurde bei dMMR-Patienten durch die NAC im Vergleich zur Kontrolle nicht signifikant gesenkt (HR 0,86, *p* = 0,68) im Gegensatz zu Patienten mit pMMR-Tumoren (HR 0,69, *p* = 0,043).

## Schlussfolgerungen der Autoren

Aus Sicht der Autoren ist die präoperative 5‑FU-basierte Chemotherapie bei operablem Kolonkrebs eine effektive Strategie, um sowohl ein präoperatives Downstaging zu erreichen als auch die Resektionsergebnisse und die Tumorkontrolle zu verbessern.

## Kommentar

In der multimodalen Therapie des Kolonkarzinoms spielte die primäre Resektion die Hauptrolle, eine adjuvante Systemtherapie wurde je nach Risikoprofil angeschlossen. Die aktuellen Entwicklungen in der Therapie des Kolonkarzinoms lassen Analogien zur früheren Entwicklung der Therapiesequenzen beim Rektumkarzinom vermuten: Dort ist die neoadjuvante Radiochemotherapie bei lokal fortgeschrittenen Tumoren seit Jahren Standard [1] und erlaubt durch optimierte neoadjuvante Schemata im Rahmen der sogenannten „totalen neoadjuvanten Therapie“ (TNT) mittlerweile sogar ein organerhaltendes Vorgehen für selektierte Patienten [2]. Aber auch ohne Hinzunahme einer Strahlentherapie kann durch eine präoperative Chemotherapie eine Verbesserung des tumorfreien Überlebens erreicht werden, wie die Daten der PROSPECT-Studie zeigen [3]. Die dort beobachteten Vorteile einer neoadjuvanten Therapie, wie präoperatives Downstaging oder eine verbesserte Krankheitskontrolle durch eine frühe Adressierung von (mikroskopischer) systemischer Tumoraussaat, lassen sich möglicherweise auf das Kolonkarzinom übertragen. Demgegenüber sollten jedoch auch die Risiken der Hinzunahme einer neoadjuvanten Therapie in der zukünftigen Evaluierung berücksichtigt werden: Insbesondere dürfte das Risiko einer Übertherapie bei Überschätzung des klinischen Tumorstadiums vor Therapie von großer Relevanz sein. Prinzipiell besteht ein Risiko für postoperative Komplikationen aufgrund der zytostatischen Therapie vorab, wobei sich diese Annahme in der FOxTROT-Studie nicht bestätigte.

Für dMMR-Patienten wird die adjuvante Chemotherapie im UICC-Stadium II aktuell aufgrund des fehlenden „benefit“ sowie der guten Prognose nicht empfohlen [4, 5]. Ähnliches scheint entsprechend den vorliegenden Ergebnissen auch für die neoadjuvante Chemotherapie zu gelten. Folglich ergibt sich die Frage, inwiefern auch bei der neoadjuvanten Therapie eine Differenzierung zwischen dMMR und pMMR bei der Indikationsstellung zu treffen wäre. Perspektivisch könnte für dMMR-Tumoren die Immuncheckpointblockade eine bessere Option sein. Erste Studien zeigen hier eine außergewöhnliche Tumorregression bis hin zur Komplettremission [6], sodass für diese Subgruppe sogar die alleinige definitive Immun(chemo)therapie denkbar wäre.

### Fazit

Die Autoren um Morton et al. konnten in dieser Phase-III-Studie erstmals trotz der Kürze der NAC eine beachtliche Verbesserung des Downstagings und der Tumorkontrolle bei guter Verträglichkeit zeigen. Die Ergebnisse unterstreichen die Bedeutung einer modernen multimodalen Behandlungsstrategie, die medikamentöse Therapie und chirurgische Intervention kombiniert, und legen eine Grundlage für die Etablierung der NAC auch beim Kolonkarzinom.

*Georg W. Wurschi, Jena*
